# Oxidised cellulose-based reaction mimicking a suspicious ovarian mass: a case report and a systematic review

**DOI:** 10.52054/FVVO.16.2.015

**Published:** 2024-06-28

**Authors:** R Nasir, I Alkiumi, E Alzahmi, B AlMaamari, H Gharbi, Z Hakim, P Koninckx, A Wattiez

**Affiliations:** Obstetrics and gynaecology department, Latifa Women and Children Hospital, Dubai, UAE; Prof emeritus Obstetrics and Gynecology KULeuven, Leuven, Belgium, the University of Oxford, Oxford, UK, University Cattolica, Rome, Italy

**Keywords:** Hemostatic agent, Surgicel reaction, suspicious ovarian mass, regenerated oxidised cellulose, Surgicel

## Abstract

Oxidised regenerated cellulose was introduced 60 years ago to control diffuse bleeding from large surfaces. Although considered safe and effective, foreign body reactions can mimic suspicious masses in several organs. We describe the third case, reported in PubMed, of an oxidised regenerated cellulose-based granuloma mimicking a suspicious ovarian tumour on MRI. During surgery, the diagnosis was suspected by granulomatous tissue and confirmed by pathology. The follow-up after the excision was uneventful. Although a rare complication, physicians should be aware of this presentation and of the recommendation to remove excess Surgicel after the bleeding has stopped.

## Introduction

Oxidised cellulose is a biodegradable and biocompatible derivate of cellulose, which is used to control diffuse capillary, venous, or small arterial bleeding when conventional methods are impractical or ineffective (Zhang et al., 2020). Biodegradation of oxidised cellulose to non- toxic materials begins within 24-48 hours and is completed in 1-2 weeks (Albala et al., 2015), varying with the degree of polymerisation. Surgicel is considered safe, although swelling and the acidic pH may cause nerve damage if applied nearby. The mechanism of action is the acceleration of hemostasis by extracting blood components and forming a gel-like pseudo-clot, which is a matrix for fibrin deposition.

These characteristics explain the indications and contraindications of regenerated oxidised cellulose (Ethicon, 2023). Surgicel should not be used for major bleedings, non-hemorrhagic serous oozing, close to nerves, or bone defects. Oxidised cellulose was initially used as sheets with a controlled thickness and later as a sprayable powder, with the recommendation not to use it on dry surfaces and to remove excessive material as much as possible to avoid granuloma formation. Although very rare, some 250 cases of granuloma formation were reported in different organs including the thyroid (Alameer, 2023; Liberale et al., 2023), the mediastinum (Zuccatosta et al., 2022; Franceschini, 2023), lung cancer (Cimenoglu et al., 2024), intracranially (Franceschini, 2021; Loh et al., 2022), in a temporo-mandibular joint (Azami Hassani and Slimani, 2021), after cholecystectomy (Spaziani et al., 2018), bowel surgery (Salmo et al., 2010; Wang and Chen, 2013), renal surgery (Sabino et al., 2007; Agarwal et al., 2010), cartilage grafts (Calvert et al., 2006), cardiac surgery (Ibrahim et al., 2002), and after ovarian surgery (Gao et al., 2002; Cormio et al., 2016), occasionally many years after surgery (Loh et al., 2022). These granulomas invariable cause diagnostic difficulties, especially after cancer surgery, often mimicking a malignant process or an abscess (Young et al., 1993).

Ovarian granulomas of Surgicel are rare, and only 2 case reports were found in Pubmed. We describe a third case in a young woman with a suspicious ovarian mass one year after laparoscopic ovarian cystectomy.

## Case Report

**Figure 1:**
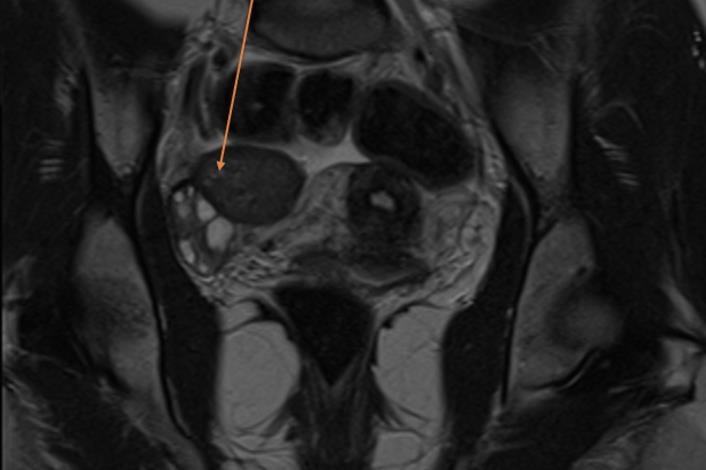
MRI appearance of ovary.

**Figure 2:**
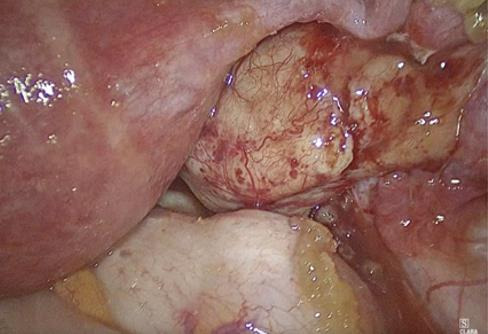
Appearance of lesion during laparoscopy.

**Figure 3:**
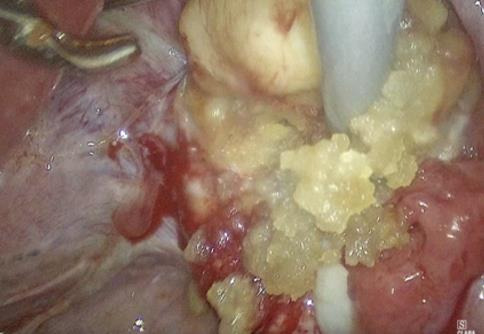
Lesions contents.

A 26-year-old woman was referred from another hospital with the suspicion of an ovarian malignant tumour because of the MRI describing a right 3.7cm ovarian cyst with an exophytic lesion (Figure 1). This occurred one year after an emergency laparoscopy for pain where an ovarian cystectomy was performed, during which Surgicel was used. Clinically, the patient did not feel unwell, had no pain, and had normal infectious parameters (WBC of 7.3). CA-125 was normal, and CA-19 was 50. Ultrasound imaging was not performed, but she underwent a laparoscopy due to the suspicious ovarian mass on MRI. A 3cm encapsulated mass was found attached to the medial aspect of the right ovary (Figures 2 and 3), but without suspicious lesions on the peritoneum, diaphragm, omentum, or appendix. The left ovary was normal. Peritoneal fluid was collected for cytology. During the dissection of the lesion from the otherwise normal-looking ovary, a thick, yellow, gelatinous material became visible (Figure 3). Suspecting a Surgicel granuloma, the ovary was left intact. The patient was discharged the next day, and the follow-up was uneventful. The histopathology confirmed our suspicion of a Surgicel granuloma, described as fragments of tissue lined by layers of foamy histiocytes, multinucleated giant cells, and inflammatory cells, with many acellular basophilic non-refractile material in between, and features suggestive of xanthogranulomatous reaction to foreign material.

## Review

Pubmed was searched for (“regenerated oxidised cellulose” OR Surgicel) AND (ovarian OR ovary), and eight records were found. These were hand-screened to find two reports of Surgicel granulomas in the ovary. One case report described a woman with pain in the left lower quadrant one month after a hysterectomy and right salpingo-oophorectomy for hemoperitoneum secondary to a ruptured corpus luteum. An 8-cm left ovarian mass with a microcystic surface was diagnosed by pathology as a massive foreign-body granulomatous reaction to Surgicel™ (Gao et al., 2002). The second case report described a woman presenting with irritative voiding six months after a cystocele repair and sling procedure, during which Surgicel was used and in whom a large Surgicel granuloma was later found. (Cormio et al., 2016)

## Discussion

This third case report of an ovarian Surgicel granuloma confirms that Surgicel can result in masses mimicking malignancy or abscesses, as has been reported over 200 times in many types of surgery. Unfortunately, the pathophysiology of these granulomas is not clear. Although delayed resorption or specific reactions in some patients cannot be excluded, we rather speculate that the use of powder instead of sheets may result in excessive material. Although contra-indicated, some surgeons might be tempted to use more Surgicel if bleeding does not stop immediately, and they might be reluctant to wash off or remove excess Surgicel, fearing to reactivate the bleeding. This cannot be proven without controllable information such as a video registration. However, it remains important to realise that some of these granulomas could have been prevented.

Surgicel ovarian granulomas are not a major problem since most women are asymptomatic, and only some present with pain (Zhang et al., 2015). However, they risk inadequate or over-treatment because of the suspicion of a malignancy or an abscess by imaging. Therefore, the surgeons’ awareness of this rare pathology is important. Imaging by ultrasound, CAT scan, or MRI is not specific and describes only a heterogeneous mass or collection containing occasionally small gas locules (Zhang et al., 2015). This is important since gas in the operative site by computer tomography, often signaling an abscess, might risk inadequate or over-treatment, although it can be a normal finding after the application of oxidised regenerated cellulose (Arnold and Sodickson, 2008).

Control of bleeding and hemostasis during ovarian surgery starts with a correct surgical technique identifying the cleavage planes and avoiding bleeding from the hilum. Unfortunately, comparative data on hemostasis are limited. Bipolar coagulation balances excessive coagulation and ovarian damage versus inadequate haemostasis and haematoma formation. Suturing risks compression of ovarian tissue with ovarian damage. Fibrin sealants are not available in many countries. Surgicel is used occasionally in ovarian surgery, although poorly investigated. Topical haemostatic agents could reduce operative time, blood loss and ovarian damage, but solid comparative data are not available. RCTs did not demonstrate a difference between bipolar electrocoagulation and the use of a hemostatic agent (Ito et al., 2018). Ovarian damage during cystic ovarian endometriosis surgery might be less when a hemostatic agent is used instead of suturing (Lim et al., 2021). However, when haemostatic agents are used, surgeons should adhere strictly to the recommendations of removing excess Surgicel before closing the abdomen. Unfortunately, it is not clear whether the IOTA criteria are helpful in the diagnosis of granulomas (Timmerman et al., 2000).

## Conclusion

This case emphasises that granulomas can occur after the use of Surgicel and that the recommendations for use should be followed strictly, especially removing excess material. In women with a suspicious ovarian mass after the use of Surgicel, a Surgicel granuloma should be considered. The available data do not permit to recommend a wider use of Surgicel.
